# Investigating the dispersal of macro- and microplastics on agricultural fields 30 years after sewage sludge application

**DOI:** 10.1038/s41598-022-10294-w

**Published:** 2022-04-16

**Authors:** Collin J. Weber, Alexander Santowski, Peter Chifflard

**Affiliations:** Department of Geography, Philipps-University Marburg, Deutschhausstr. 10, 35037 Marburg, Germany

**Keywords:** Environmental monitoring, Environmental impact

## Abstract

Plastic contamination of terrestrial ecosystems and arable soils pose potentially negative impacts on several soil functions. Whereas substantial plastic contamination is now traceable in agro-landscapes, often internal-caused by the application of fertilizers such as sewage sludge, questions remain unanswered concerning what happens to the plastic after incorporation. Based on a combined surface and depth sampling approach, including density separation, fluorescence staining and ATR-FTIR or µFTIR analyses, we quantified macro- and microplastic abundance on two agricultural fields—34 years after the last sewage sludge application. By sub-dividing the study area around sludge application sites, we were able to determine spatial distribution and spreading of plastics. Past sewage sludge application led to a still high density of macroplastics (637.12 items per hectare) on agricultural soil surfaces. Microplastic concentration, measured down to 90 cm depth, ranged from 0.00 to 56.18 particles per kg of dry soil weight. Maximum microplastic concentrations were found in regularly ploughed topsoils. After 34 years without sewage sludge application, macro- and microplastic loads were significantly higher on former application areas, compared to surrounding areas without history of direct sewage application. We found that anthropogenic ploughing was mainly responsible for plastic spread, as opposed to natural transport processes like erosion. Furthermore, small-scale lateral to vertical heterogeneous distribution of macro- and microplastics highlights the need to determine appropriate sampling strategies and the modelling of macro- and microplastic transport in soils.

## Introduction

Plastic contamination is nowadays an emerging threat to all ecosystems and environments worldwide. Especially microplastics, as solid and insoluble particles with a defined size smaller 5 mm^1,2^ occur widespread through different ecosystems, including remote mountain areas to highly urbanized landscapes^3–6^. Over the past decade the evidence of plastic contamination of terrestrial ecosystems including arable land and agricultural soils has been provided^7–10^. Different studies suggest the high abundance of plastics and microplastics in agricultural soils, with major plastic hotspots in topsoil often caused by “plasticulture” or the application of plastics in agriculture^7,8,11^.

Worldwide arable land covers over 47 million km^2^ or 10.8% of global land area^12^, but is subject to various threats leading to soil degradation^13^. Since 95% of global food is directly or indirectly produced via land-based agriculture on soils, further soil degradation will affect global food availability and food security^14^. This global issue is enhanced by increasing population growth, and ultimately advancing climate change^13^. Besides all well-known threats affecting terrestrial ecosystems and soils, plastic contamination could also lead to further degradation of soils^15,16^. A wide range of studies have shown that the presence of plastics and microplastics in soils could negatively affect soils properties, soil organisms or plant growth^17–21^. Furthermore, microplastic uptake by plants and introduction into the food chain^15,22–25^ can pose potential health problems in humans^26,27^.

All of these influences are strongly related to arable land and agricultural soils as the hub of global food production. In addition to the indirect emission of plastic and microplastics into arable soils as a result of wind or water transport through littering, agricultural practices themselves systematically generate a major source of plastic emissions in soils^28,29^. The main sources of emissions were identified as plastic contaminants from fertilizers such as compost, digestate or sewage sludge, which are directly and regularly introduced into soils for the purpose of soil improvement and fertilization^7,28,30^.

The use and release of sewage sludge from wastewater treatment with its high organic and nutrient content, but also foreign matter such as plastic, represents a major pathway for the anthropogenic redistribution of plastic residues from aquatic to terrestrial systems^31–33^. Sewage sludge itself was found to contain on average, 14.750 particles per kilogram, or 97.66 microplastic particles per gram^32,33^. Initial studies focussing on plastic contamination of soils caused by sewage sludge application indicate that sewage sludge application led to high concentrations of microplastic in topsoil, in contrast to soils not fertilized with sludge^8,31,33^. Aware of the foreign matter content, as well as other contaminants in sewage sludge, such as heavy metals and organic pollutants, sludge application is often subject to meeting certain legal requirements. For example, in Germany, sludge application is limited to 5 tonnes (t) per hectare (ha.) every three years^34^. Despite the available sludge resources, the agricultural utilization of sewage sludge is continuously declining in Germany, and will likely be further reduced by increasing regulations in the future^34^.

Despite new regulations, large amounts of sewage sludge, and associated plastics, have already been systematically introduced into soils in the past. Even though this emission pathway and the occurrence in soils are already known, questions still remain as to what happens to the plastic from sewage sludge in arable soils over longer periods of time? In particular, the question arises: how is plastic distributed spatially (laterally and vertically) by natural transport processes (e.g., soil erosion), as well as by the technical treatment of the agricultural area (e.g., ploughing)?

Here we investigate macro- and microplastic abundance, and its spatial distribution, on two agricultural fields in Central Germany, located within an area that is affected by recent microplastic contaminations from sewage sludge^28^. Studying former sewage sludge application areas and their surroundings, we investigate whether macro- and microplastics remain at their original application area, or become distributed more widely over time. The research area is one which is managed under controlled conditions—as part of the agricultural teaching and research unit “Rauischholzhausen”, with known management data for recent years. Both fields were part of a fertilizer trial involving application of sewage sludge from 1969–73 to 1986^35^, and were subsequently farmed conventionally, without further application of sewage sludge, compost or plasticulture. Our study represents a first attempt to describe the lateral and vertical spatial occurrence and spreading of macro- and microplastics from old sewage sludge applications on agricultural fields.

## Results

### Plastics on soil surfaces

**Figure 1:**
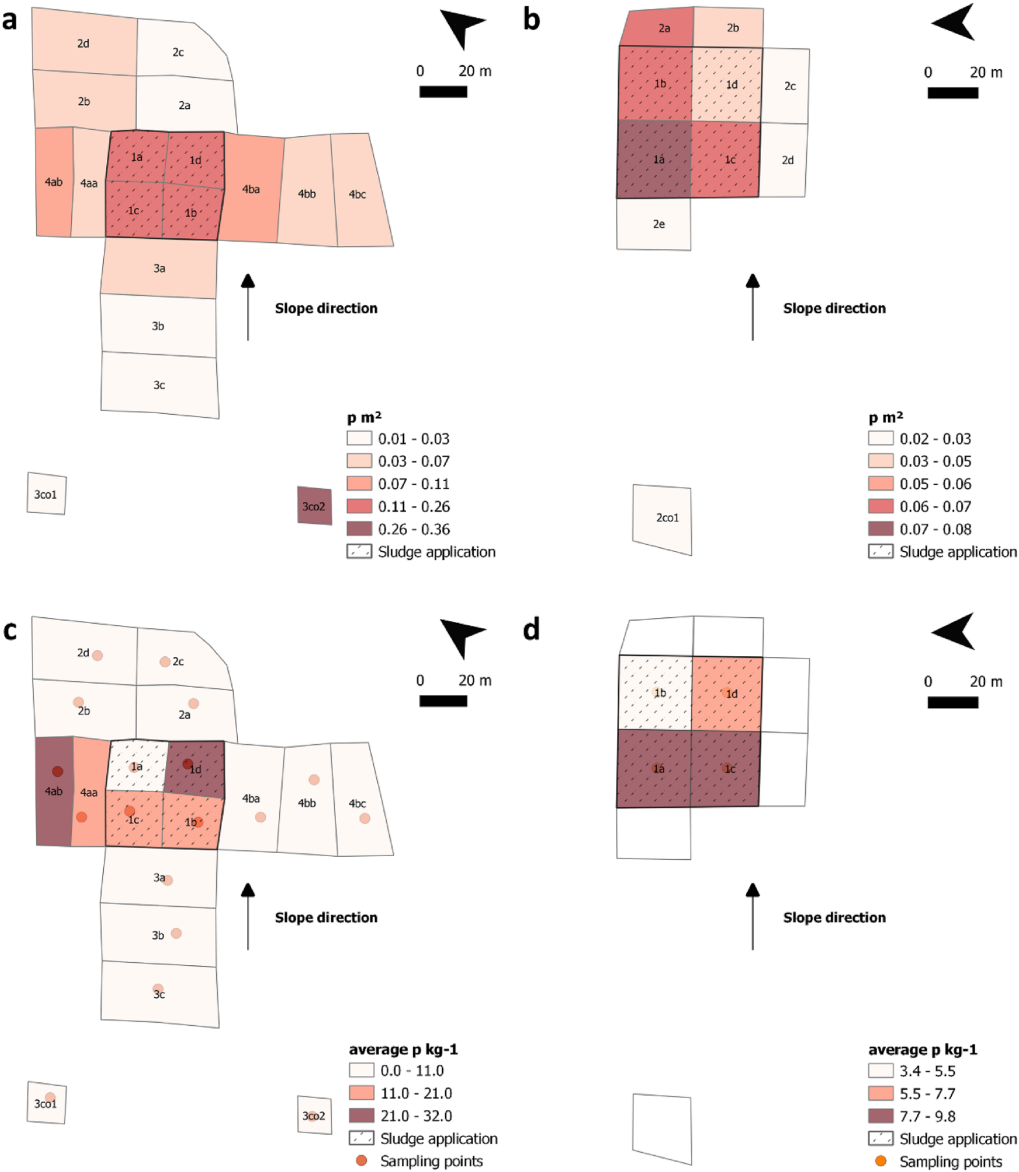
Surface plastic concentration (p m^2^) on agricultural field (**a**) “Lehmkaute” (LK) or (**b**) “Holzbach” (HB) and average microplastic concentration (p kg^-1^) over entire soil column in agricultural field (**c**) “Lehmkaute” and (**d**) “Holzbach” (Overview of sampling sectors and points based on a digital terrain model provided in Supplementary Fig. S1).

The two agricultural fields, with a total investigated area of 2.18 ha, yielded 1389 macroplastic particles (> 5 mm), resulting in a total plastic abundance of 637.12 p ha^−1^. The two agricultural fields were subdivided into 18 (field LK) and 10 (field HB) study sectors, allowing for the determination of spatial differences in plastic occurrence (Fig. 1a,b).

**Figure 2:**
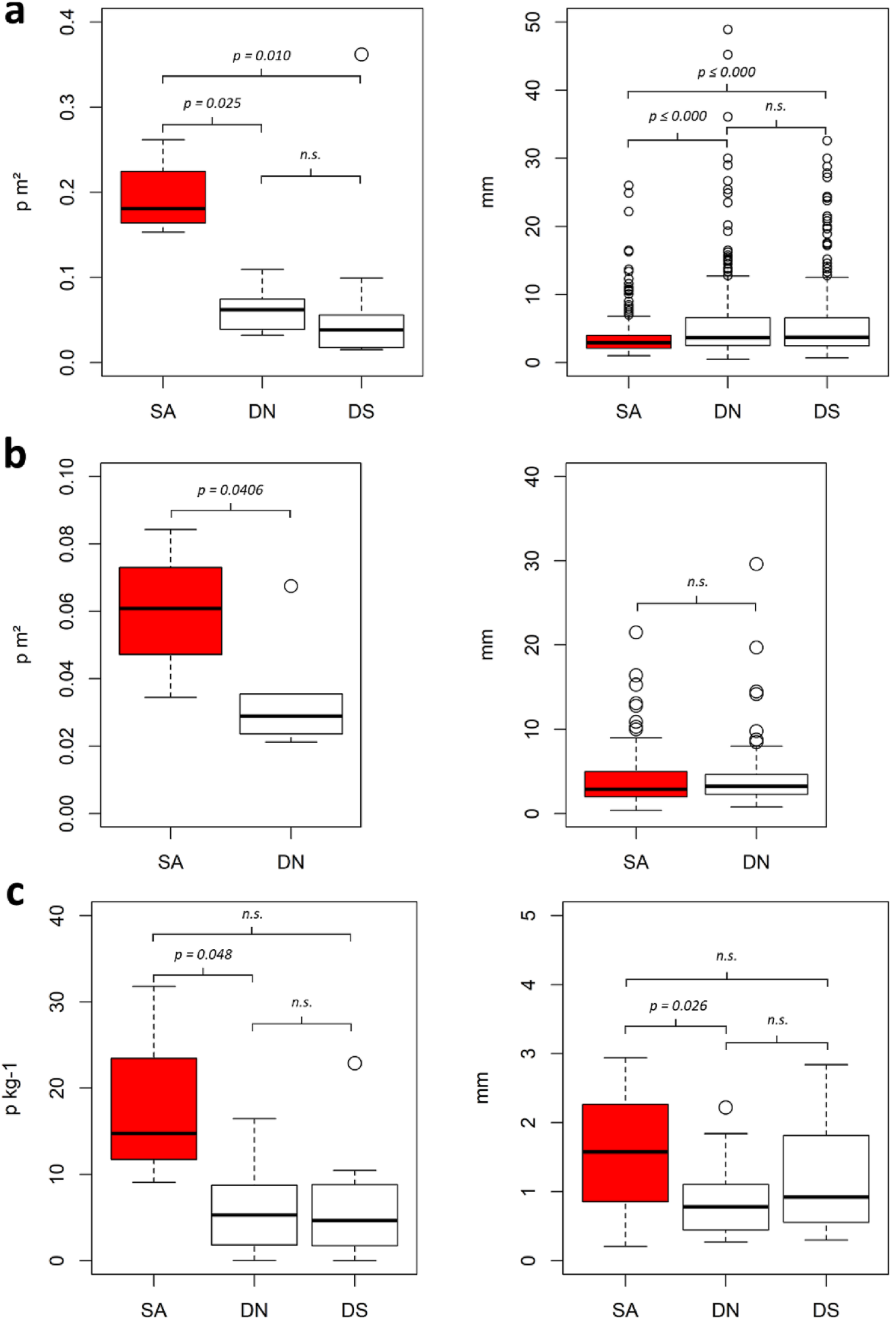
Differences of plastic concentrations and particle sizes between sampling sectors. With (**a**) macroplastics on soil surfaces on field “Lehmkaute” and (**b**) field “Holzbach”. (**c**) Microplastics in soils of field “Lehmkaute”. Grouped sectors: (SA) sludge application sectors, (DN) direct neighbour sectors and (DS) distant sectors including control sectors. Significant mean differences (*p* ≤ 0.05) signed and insignificant differences expressed through n.s.

On both fields, the highest concentration of plastic items was found within the sectors representing the former sewage sludge application area, with average values of 0.19 p m^2^ for field LK and 0.06 p m^2^ for field HB. Within the former sewage sludge application areas, the concentrations vary in the range of 0.15–0.26 p m^2^ (LK) and 0.03–0.08 p m^2^ (HB) (Table 1). Grouping sectors to sewage sludge application area (SA), direct neighbour sectors (DN) and more distant sectors (DS) shows statistically significant (*p* ≤ 0.05) differences in plastic concentration (Fig. 2).

**Table 1:** Plastic concentrations on soil surfaces and microplastic concentrations in soils according sampling depth and sampling sectors.

Field	Sectors	Position^a^	Plastic load at soil surface			(Micro-)plastic loads at depth (p kg^−1^)			
			Number of particles	Plastic load (p m^2^)	Plastic load (p ha^−1^)	Average	0–30 cm	30–60 cm	60–90 cm
LK	1a	SA	96	0.17	1744.82	9.07	9.57	0.00	17.63
	1b	SA	89	0.15	1530.79	15.11	9.15	13.25	22.95
	1c	SA	111	0.19	1871.21	14.40	30.50	0.00	12.70
	1d	SA	154	0.26	2617.27	31.80	56.18	13.76	25.45
	2a	DN	32	0.03	321.32	8.77	14.96	0.00	11.34
	2b	DN	66	0.06	621.41	1.83	5.49	0.00	0.00
	2c	DS	13	0.02	153.46	1.58	4.75	0.00	0.00
	2d	DS	62	0.05	532.69	10.46	31.37	0.00	0.00
	3a	DN	48	0.04	389.29	8.46	19.04	6.34	0.00
	3b	DS	22	0.02	177.11	1.74	5.23	0.00	0.00
	3c	DS	21	0.02	164.08	2.07	6.22	0.00	0.00
	3co1	Control	5	0.02	183.82	5.66	9.49	7.50	0.00
	3co2	Control	8	0.36	3619.91	0.00	0.00	0.00	0.00
	4aa	DN	51	0.07	744.96	16.46	23.00	13.57	12.81
	4ab	DS	74	0.10	994.89	22.89	36.52	6.78	25.36
	4ba	DN	125	0.11	1094.09	2.13	0.00	0.00	6.40
	4bb	DS	60	0.06	558.40	8.83	0.00	20.13	6.34
	4bc	DS	38	0.04	383.26	4.65	6.83	0.00	7.11
HB	1a	SA	74	0.08	842.43	9.82	18.41	11.04	0.00
	1b	SA	57	0.06	617.45	3.35	0.00	10.06	0.00
	1c	SA	50	0.06	598.21	8.09	5.25	14.05	4.97
	1d	SA	29	0.03	345.32	5.99	5.25	14.05	4.97
	2a	DN	28	0.07	674.38	Not sampled, see “Soil sampling” section for further information			
	2b	DN	16	0.04	355.65				
	2c	DN	12	0.02	212.46				
	2d	DN	15	0.03	269.15				
	2e	DN	15	0.02	236.41				
	2co1	Control	18	0.03	310.35				

^a^Position on agricultural fields: Sludge application area (SA), direct neighbours to SA (DN), distant sectors (DS) and control sectors (control).

Surface plastic concentrations at field LK was 67.3% higher at the sewage sludge application area (mean = 0.19 p m^2^), compared to direct neighbour sectors (mean = 0.06 p m^2^) and similarly significantly higher compared to distant sectors (Mean = 0.08 p m^2^). At field HB, average surface plastic concentration at the sewage sludge application area (mean = 0.06 p m^2^) exceed direct neighbour sectors (mean = 0.03 p m^2^) by 42.9%. Control sectors showed comparatively low concentrations, but not the lowest, with one occurrence of a high plastic concentration of 0.36 p m^2^ at control sector 3–2 of field LK.

Plastic concentration was not correlated with terrain elevation, possibly due to the limited slope of both fields (Table 2). On the other hand, the distance of each sector from the centre of the sludge application area was correlated to plastic concentration, indicating a statistically significant the decrease in surface plastic concentrations with increasing distance from sewage sludge application areas on both fields.

**Table 2:** Spearman coefficients r_Sp_ of correlations between plastic loads (surface and depth sampling) and terrain height or distance to sewage sludge application area.

Field	Plastic loads	Variables^a^	r_SP_	*p*-value	Significance^b^	*n*
LK	Surface plastic load (p m^2^)	A	0.12	0.6325	n.s	18
		B	− 0.49	0.0417	*	18
HB		A	− 0.49	0.1544	n.s	10
		B	− 0.65	0.0490	*	10
LK	Average MP load (p kg^−1^)	A	− 0.24	0.3350	n.s	18
		B	− 0.65	0.0046	*	18
	0–30 cm MP load (p kg^−1^)	A	− 0.15	0.5581	n.s	18
		B	− 0.52	0.0255	*	18

^a^A: Surface height at central sector point (m a.s.l.), B: Distance to central sewage sludge application area (m).

^b^Significant * if *p* ≤ 0.05, n.s = not significant.

Average plastic item size (combined field range: 0.4–57.5 mm) was 5.95 mm on field LK, and 4.08 mm on field HB respectively. While we found significant differences between item sizes on field LK between sewage sludge application area (mean = 3.47 mm) and direct neighbour sectors greater (mean = 5.94 mm), the size distribution between sewage sludge application area (mean = 3.97 mm) and surrounding sectors (mean = 4.08 mm) on field HB is comparable (Fig. 2).

With regard to the form of the plastic items, we found an overall dominance of plastic fragments (52.6% at LK and 41.1% at HB) and films (41.0% at LK and 50.3% at HB), followed by other plastic formations (6.4% at LK and 8.6% at HB) as well as identifiable pieces of cable ties, filaments, pellets and styrofoam. The surface of items at field LK showed a relatively equal distribution of degradation stages: emerging alteration (36.9%), fresh (27.7%) and weathered (28.0%) surfaces. In contrast, items on field HB appeared to be predominantly weathered (89.2%) or showing emerging signs of alteration (8.3%) to its surface structure. An example of plastic items is given in Supplementary Fig. S2. Plastic item colouration was predominantly white (41.1%), and included blue (14.0%), transparent (13.5%), black (7.8%) coloured plastic, in addition to smaller amounts of green, grey, orange, pink, red and yellow, as well as multi-coloured fragments.

**Figure 3:**
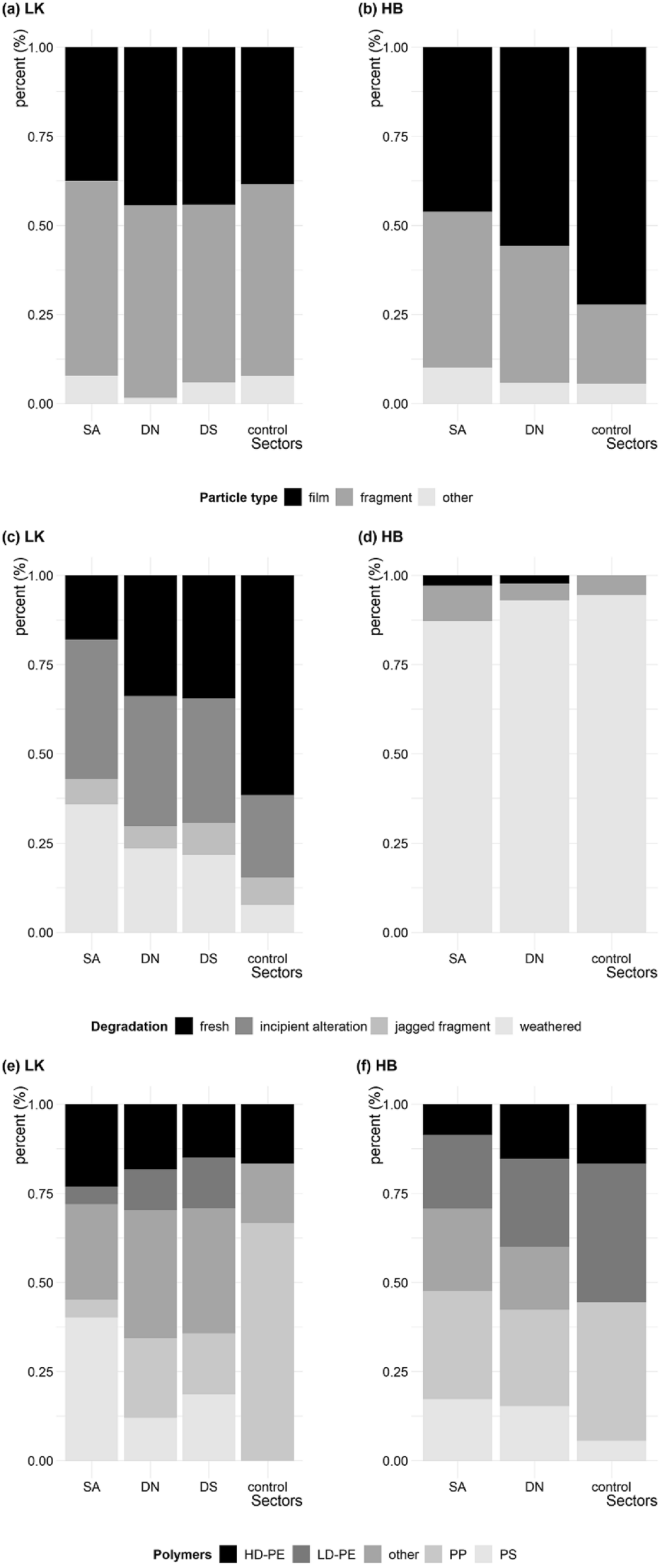
Characteristics of plastic items from soil surfaces in comparison between sector groups: (SA) sludge application sectors, (DN) direct neighbour sectors and (DS) distant sectors including control sectors. Item form composition on field (**a**) “Lehmkaute” and (**b**) “Holzbach”, item surface degradation composition on field (**c**) “Lehmkaute” and (**d**) “Holzbach”, item identity composition on field (**e**) “Lehmkaute” and (**f**) “Holzbach”.

We found notable differences in the form of plastic at each field site. At field HB there was a higher proportion of films, while fragments dominated at field LK. There was a substantially higher overall percentage of weathered particles at field HB compared to LK, which was predominantly fresh or emergent weathering, with a clear pattern of increasing degree of fresh (un-weathered) plastic, with increasing distance away from the application area and toward the control sections (Fig. 3).

Item identity, based on ATR-FTIR analyses showed a dominance of common polymers such as polystyrene (PS), polypropylene (PP), low- or high-density polyethylene (LDPE, HDPE) and others like polyethylene terephthalate (PET). The aforementioned common polymers accounted for 74.8% of the composition on plastic at field LK, and 79.9% at field HB. The share of polymer composition, especially within other polymers, including polyvinyl chloride (PVC), polysulfone (PES), polycaprolactam (nylon 6) or synthetic resins, showed substantial variation among the different sector groups (Fig. 3c).

### Microplastics within soils

Based on a combined microplastic analysis approach using density separation with NaCl solution, fluorescence identification based on Nile Red staining, and µFTIR analyses, we found 0–9 microplastic particles (<5 mm) in 40 g dry soil (mean = 1.5 particles per 40 g). Microplastic concentrations ranged from 0.00 to 56.18 p kg^−1^, with an overall average of 8.88 ± 1.21 p kg^−1^. Average plastic concentrations per study sector, and over the total sampling depth of 90 cm, ranged from 0.00 to 31.80 p kg^−1^ (Table 1). Average microplastic concentration at the sewage sludge application sites ranged from 9.10 to 31.80 p kg^−1^ on field LK (Fig. 1c), with lower concentrations of 3.35–9.82 p kg^−1^ on field HB (Fig. 1d). Due to the sampling regime, comparisons between sector groups can be made for field LK. Average microplastic concentrations on the sewage sludge application area was notably higher (67.5–83.9% higher) than the surrounding sectors, with the exception of sector 4 (Fig. 1c). Average microplastic concentrations were not statistically significant between the sewage sludge application area (av. 17.59 p kg^−1^) and its direct neighbour sectors (mean = 7.53 p kg^−1^), but were significantly different from distant sectors (mean = 4.37 p kg^−1^) and control sectors (mean = 2.83 p kg^−1^) (Fig. 2c).

As in the case of surface plastic concentrations, mean microplastic levels across depth and concentration at 0–30 cm (plough horizon) significantly and negatively correlated with distance of sampling points from the centre of the sludge application area (Table 2).

Sampling at three different soil depths, revealed a clear accumulation of microplastics in the upper 30 cm of soil (plough horizon) on field LK. Average microplastic concentrations were significantly higher (ANOVA, *p* = 0.0070) in uppermost soil section (0–30 cm; mean = 14.91 p kg^−1^) compared to that at 30–60 cm depth (69.7%; mean = 4.52 p kg^−1^) and at 60–90 cm depth (44.8%; mean = 8.23 p kg^−1^), At field HB, we found higher concentrations in the mid soil sections (mean = 12.30 p kg^−1^), compared to the uppermost (mean = 7.23 p kg^−1^) or lowest (mean = 2.48 p kg^−1^) sections.

Overall, we found microplastics in a size range of 0.3–4.91 mm, with an average length of 1.38 mm and a total of four identified particles below the method size limit of 300 µm. We found significantly (ANOVA, *p* = 0.0007) smaller particles (mean = 0.93 mm) in the lower soil section (60–90 cm), compared to the upper soil section (0–30 cm; mean = 1.77 mm). Particle sizes in the top and mid sections (mean = 1.45 mm) were comparable. Furthermore, microplastics at the sewage sludge application area showed greater particle sizes than the direct neighbour sectors (Fig. 2c).

**Figure 4:**
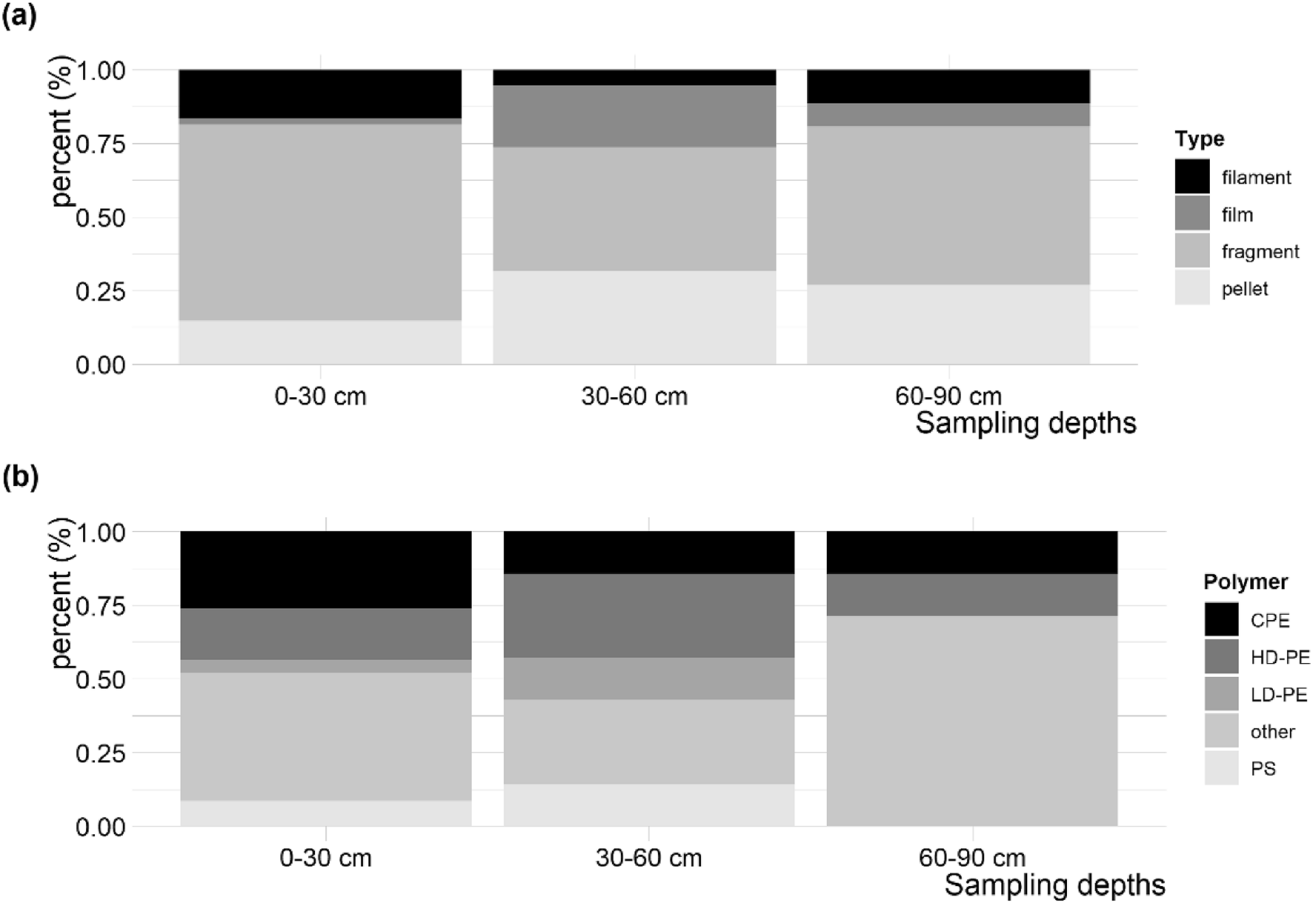
Characteristics of microplastic particles according sampling depths for (**a**) microplastic form types and (**b**) microplastic identity composition.

Microplastics occurred mainly as fragments (58.6%), followed by pellets (21.2%), filaments (13.1%) and films (7.1%), with overall incipient alteration or weathered surface structures. Polymer composition based on µFTIR analyses shows dominance of chlorinated polyethylene (CPE, 21.6%), rubbers (21.6%) and high-density polyethylene (HDPE, 18.92%) among others. Microplastic type and polymer composition differed between sampled soil depths (Fig. 4). Clear differences in shape, degradation status or polymer composition between sector groups were not apparent—indicating an even character of plastic throughout the different sector groups.

## Discussion

### Plastics on soil surface

Even though larger plastic debris, which can be described as macroplastics, is visible almost everywhere in the environment (e.g., roadsides, urban areas, streams, canals), to the best of the authors knowledge, there is only one published study which investigates surface plastic loads on agricultural soils. Since the study by Piehl et al. (2018) was exemplary for ours, the results obtained can be compared well: They report an overall load of 206 p ha^−1^ on a field not affected by sewage sludge or other plastic containing fertilizers^29^. Therefore, soil surface macroplastic load can be estimated more than three times higher on fields with sewage sludge application, expressed through the overall load of 637 p ha^−1^ found on our study fields. Plastic item sizes of both studies were within a comparable range between >5 mm and approx. 50 mm. Furthermore, plastic item identity in both studies predominantly comprised of common polymers, which are mass produced and used^36,37^. Many plastic items on soil surfaces could be identified as fragments from items for packaging purposes (e.g., Supplementary Figs. S1 and S2). This suggests that especially everyday-life products end up in sewage sludge or agricultural soils, especially in the case of waste sewage sludge application like on field LK.

In general, it can be concluded that higher sludge application levels led to higher microplastic loads as the field “LK” showed an average load of 0.19 p m^2^ after an application of 120–200 t ha^−1^ (1973–1986, three-year interval) and the field “HB” only 0.06 p m^2^ after an application of 2.5–5.0 t ha^−1^ (1969–1986, three-year interval). This finding is also supported by other studies on microplastics in sewage sludge influenced soils^8,31–33^.

In contrast to former studies, the special setting of our study fields, with relatively “old” sewage sludge applications (>30 years) and the application only on a small area with the same cultivation practice of the total field areas, enables us to examine spatial distribution in a different manner. We found a clear evidence that soil surface macroplastics loads occur in significantly higher contents on former sewage sludge application areas than on surrounding, non-treated field areas. These led to the assumption that the majority of plastic contaminations stay spatially stable on application areas whereby only a minor portion is further dispersed. In addition to the disparity in surface macroplastic loads, we also found that larger items tended to be found outside the former application area. This finding could indicate that greater items could be easier spread or that items staying stable on sludge application area becoming more degraded through e.g., physical degradation from ploughing^38,39^.

With regard to spatial spreading of macroplastic contaminants over an agricultural field, transport processes by wind ^33,40^, and water ^41^ or anthropogenic activity (e.g., ploughing) are likely. We could not find any evidence for redistribution by wind, especially since many items were partly covered with soil during sampling, and are therefore heavy. For a directional redistribution, downslope by water erosion, we could also not find any evidence, like an accumulation at the foot slope or a data correlation with the terrain height^41,42^. The decrease in plastic content with increasing distance from the application area, as well as the higher content in sectors parallel to the slope, could indicate that distribution takes place primarily through tillage and ploughing, against a background of >30 years of continuous cultivation after the last sludge application.

Our sampling and study design cannot be directly transferred to other arable fields, as these are usually managed and fertilized uniformly per field. Nevertheless, it can be assumed that uneven application of sewage sludge and distribution by ploughing will also result in heterogeneous distribution patterns of plastic contaminants. This may also explain the heterogeneous distributions found for microplastics in arable topsoils^8,9,43^. Those heterogenous distribution patterns may also be forced by soil erosion, if inclination is sufficient. On the other hand, it is clear that plastic residues persist for long periods >30 years, stable in position with little tendency to displacement. Against the background of the assumed long residence time of plastics under buried conditions (e.g., shielding from UV light) and despite physical influences (e.g., degradation by abrasion) our findings indicate that soils will probably be exposed to macroplastic deposits and therefore potential microplastic deposits with continuing degradation in future, for a considerable prolonged period of time^39^.

### Microplastics within soils

Unlike macroplastic loads and the limited comparability with other studies, the levels of microplastics can be compared well to other investigations in the meantime, considering methodological differences. Based on our microplastic analysis, including density separation with sodium chloride (separation of particles with a density ≤1.20 g cm^3^) and Nile Red staining identification, we found loads of 0.00–56.18 p kg^−1^ (av. 8.88 ± 1.21 p kg^−1^) in the size range of 0.3–4.91 mm. Compared to microplastic levels in agricultural soils without sewage sludge application or other use of agro-plastics, the microplastic loads are distinctly higher. Piehl et al. (2018) report loads of 0.34 ± 0.36 p kg^−1^ for agricultural topsoil (visual identification, >1 mm). In contrast, the microplastic levels we found are clearly lower than in agricultural soils with recent sewage sludge or agro-plastics application. For example, Liu et al. (2018) report average concentrations of 70.0 ± 12.91 p kg^−1^ (5.0–0.02 mm plastics) and Zhang and Liu (2018) an average of 18,760 p kg^−1^ (10.0–0.5 mm plastics), which exceeds the contamination level found here several times. Furthermore, concentrations of 0.6–10.4 p g^−1^ (<2.0 mm plastics) found by Corradini et al. (2019), maximum of 14.6 p g^−1^ (>100 µm plastics) found by Tagg et al. (2022), 320.0 ± 112.2 p kg^−1^ (> 0.25 mm) found by Ragoobur et al. (2021) or 2130 ± 950 p kg^−1^ (50–5000 µm light density plastics) found by van den Berg et al. (2020) are all considerably higher. The differences in concentration may be due to methodological differences, such as the consideration of smaller plastic particles, since the number of particles tends to increase as their size decreases^20,44^. However, the recent application of sewage sludge, agro-plastics or fertilizers in other studies could also lead to higher concentrations, since the agricultural plastic input was not interrupted for a longer period in these cases.

In the case of spatial distribution of microplastics, we found that microplastic contamination is higher on former sewage sludge application areas than in the surrounding area, as we also found for macroplastics on soil surfaces. The fact that sludge has a significant influence on the microplastic levels within the soils has already been demonstrated by other studies, most of which found statistically significantly higher levels on sludge application fields than on control sites^8,31,33^. Furthermore, those impacts are also evident for other agro-plastics like mulching films^7,11^. Considering microplastics on the same field and under consistent cultivation practice (annual cultivation of different products, as well as multiple plowing and cultivation), it must be noted that the majority of microplastics remain on the sludge application area and are not transported lateral over 30 years. As with the macroplastics, we found no evidence of wind transport or water transport (erosion) as other studies^33,41^, but a correlation with distance, indicating that microplastic contaminations regress lateral with increasing distance from former application area. Wind transport also seems to be unlikely since all the macroplastic residues had soil material attached to them and are therefore heavier as plastics itself. Furthermore, microplastic particle sizes are greater on sewage sludge application area than on direct neighbour sectors, and thus opposite to mesoplastic sizes on soil surfaces. It is assumed that smaller microplastic particles can be transported more easily, if transport by wind or water takes place^41,45^. Even if we found no evidence for those transport processes, the shifting of microplastic through tillage and ploughing could also be achieved differently here for smaller particles.

With regard to the vertical distribution of microplastics our results suggest show that the highest concentrations occur in topsoil (here: plough horizons, 0–30 cm). This finding goes in line with the overall occurrence of high microplastic concentrations in topsoil and heterogeneous concentrations in ploughing horizons^31–33^. In deeper soil layers the microplastic concentrations decrease, but are partwise higher in mid soil sections (30–60 cm, field HB) or rise again within lowest soil section (60–90 cm). With regard to the cultivation practice of the field, this finding could be attributed to a past deep ploughing, although a deep ploughing was not documented for the past 5 years. Such vertical distribution patterns are opposite to others, which found an overall decrease of microplastic concentrations with depth^33,46^. In general, the detection of mircoplastics in deep soil layers and the heterogeneous vertical distribution is a clear indication of vertical relocation processes. Supporting the vertical relocation, we found smaller microplastic particles in deeper soil layers than in topsoils, indicating an easier displacement or mobility of smaller particles, as already found in floodplain soils^47,48^. In fertile arable soils, besides percolation (transport through soils pore space and preferential flow paths), soil organisms can also contribute to vertical displacements^17,49^.

The composition of microplastic characteristics is not comparable to that of the macroplastics on soil surfaces. While in both cases fragments dominate as shape, we found more microplastic pellets and filaments within the soil, whereas macroplastics are dominated by films. Likewise, microplastic particles showing overall degraded surface conditions, while mesoplastics showing a share of 27.7% fresh particles. Both results and differences between soil surface and soil itself, may indicate that films are more difficult to reach the depths or degrade more slowly, due to their higher flexibility than fragments^38^. Despite these differences, shape form composition of microplastic particles is comparable to the findings of other studies^31^.

With regard to polymer composition we found that polyethylene (as chlorinated or high-density polyethylene) and rubbers are dominant, followed by common polymer types. Polyethylene is one of the most important and the second most widely produced in the European Union^36^ and also represented in the polymer share of macroplastics on soil surfaces and other studies^29^. Instead, rubbers do not occur in the macroplastic portion. This may be attributable to the fact that the share of rubbers does not originate from the sewage sludge but from the further usage of the fields, e.g., through the abrasion of tyres or agricultural equipment in general^50^.

Based on our study design, the findings of microplastic occurrence and spatial spreading, indicate heterogeneous lateral as well as vertical distribution patterns, comparable to macroplastics on soil surfaces. Transferred to other arable fields in a more general way, microplastics showing relative stable spatial locations and remain in their main proportion on application areas but can be shifted vertically and reach deeper soil sections.

## Conclusions

Sewage sludge is a distinct gateway for plastic contaminants in soils. With our study results, we demonstrated that both the macro- and microplastic contamination of agricultural soils is still high even after more than 30 years without sewage sludge application. Through the controlled spatial sampling conditions, it could be shown that meso- and microplastics remain spatially stable over longer periods of time and only parts are spatially spread out. While other studies have already shown evidence of the influence of sewage sludge on microplastic levels, we were able to indicate that human activity in particular, such as soil ploughing, contributes to a further spatial distribution across the arable field. Stable spatial conditions on the lateral and the observation of vertical relocations of microplastics into deeper soil section, indicate slow mobility on soil surfaces and through the soil after more than 30 years. This implies that even if the amount of foreign matter in or the use of sewage sludge itself, is further reduced and regulated, the use of sewage sludge to date causes a long-term plastic contamination in arable soils.

Thus, arable soils can act as reservoirs of the anthropogenic plastic contamination and new environmental regulations and plastic prevention strategies are already too late. Any known and new consequences of plastic contamination on soils, soil organisms or plants that are discovered by science, will thus have an impact over a sustained period of time. Therefore, future research should not only focus on environmental consequences, but also consider whether comparatively large macroplastic contaminations in particular could also be removed from soils or soil surfaces.

Next to this, the spatial heterogeneity indicates that measured plastic concentrations on a single arable field, can be very heterogeneous even within the field under the same management practise. Future research, but also possible plastic monitoring in the future, if plastic residues in soils are included in environmental legislation, need to consider this for a representative spatial assessment of plastic contaminations.

## Methods

### Study area

The study area is located in central Germany (Hesse) (50.760591, 8.876867), approximately 70 km north of Frankfurt, within the "Amöneburger Basin". The landscape is a predominantly cultivated basin, north-east of the tertiary low mountain range Vogelsberg. The basin landscape is surrounded by forested (tertiary basalt or sandstone) ridges, and is characterized by flat slopes with Pleistocene loess accumulations. Within the basin Luvisols, rare preserved Chernozems, Cambisols on upper slopes and Gleysols in the depressions have developed (FAO WRB taxonomy)^51^. Within the study area, two agricultural fields with a total area of 7.40 ha, at an altitude of ~ 244 m a.s.l. and an average slope of ~ 0°–9° were sampled. Both fields contain Luvisols and Stagnosols, developed in loess-rich colluvial silty or primary loess containing Ap horizons (down to 25.8 cm on average) followed by E and Bt horizons partwise with stagnic properties. Access to both sampling fields is limited by dirt roads or forest. The two fields, “Lehmkaute” (LK, area: 5.69 ha) and “Holzbach” (HB, area: 1.71 ha) were part of a fertilizer trial with sewage sludge from 1969 (HB) and 1973 (LK), to 1986 in partial plots (LK: 2313.2 m^2^, HB: 3477.2 m^2^) (Supplementary Fig. S1)^35^. Field LK shows only slight inclines, ranging from 0.1° to 2.0°, except for an edge area with slopes between 2° and 6°, while field HB is more strongly inclined—with slopes between 1° and 9°. Sewage sludge with a portion of 22.8 wt% foreign substances (e.g., plastics, glass) was applied as waste sewage sludge compost (mixture of domestic waste, sewage sludge and compost) in a three-year interval on field LK and as sewage sludge on field HB^35^. At field LK, ~ 120–200 t ha^-1^ was applied, with substantially smaller amounts ~ 2.5–5 t ha^-1^ applied to field HB^35^. Since 2016, both investigated fields were fertilized with digestate, as well as mineral fertilizers and crops including beets, wheat and corn cultivated (Supplementary Table S1). No agricultural plastics or plasticulture were applied on the fields. Both fields were ordinary ploughed along lines parallel to the natural slope, and at depths of 20–30 cm. Weather conditions after the last sewage sludge application (period 1986–2021) showing an average annual temperature of 9.7 °C, annual precipitations ums of 678.5 mm and on average 78.4 frost days from which 17.7 days show snow cover (Supplementary Table S2). Wind conditions showing average wind speeds <12 km h^-1^ and maximal gust speeds <72 km h^-1^ (Supplementary Table S2).The study complies with local and national regulations and permission was obtained for collection of samples.

### Surface sampling

Sampling of plastic items, with a focus on macroplastics (>5 mm)^1^, on soil surfaces (reported as particle per m^2^ or ha) was carried out in early December 2019, three weeks after sowing wheat with a seedling height of around 5 cm during sampling. Between last tillage (seeding) and sampling, three average rainfall events occurred, which contribute to the exposure of the items on soil surfaces. The sampling procedure followed that of Piehl et al. (2018). At each field, a margin of 5 m was left along the field borders, in order to exclude the influence of dirt roads or adjacent fields (Supplementary Fig. S1). The former sewage sludge application areas on each field were then divided into 4 sectors, and the surrounding area a divided into direct neighbour and distant sectors (Supplementary Fig. S1). Finally, at each field, one or two control sectors, with the greatest distance to the sludge application sectors, were sampled. This procedure resulted in a total study area of 1.51 ha (LK, 18 sectors, average size: 847.13 m^2^) and 0.67 ha (HB, 10 sectors, average size 667.89 m^2^). During the field survey, macroplastic items (>5 mm) were collected, walking along several transects with a distance of 0.5 m to each other directed over each sector. This allowed to examine the entire soil surface of each sector. Potential plastic items were collected from the soil surface and stored in PE bags^29^. In the laboratory, soil was washed from the collected plastic items using water, and the plastic dried (40 °C), photographed with scale and size measured, numbered and stored in PE bags for further analysis^29,52^.

### Soil sampling

Soil sampling for microplastic (<5 mm)^1^ analyses (reported as particle per kg soil dry weight) was conducted directly after surface sampling. Two drill cores (stainless-steel corer, diameter: 2 cm) were recovered from the centre of each sector. An aggregate sample of the two cores for three separate depth classes (0–30 cm, 30–60 cm and 60–90 cm) was made, by mixing the respective sections of the two soil cores with a metal spatula. Final soil samples were stored in in corn starch bioplastic bags (Mater-Bi bags, Bio Futura B.V.)^47^. Field-fresh soil samples were afterwards dried (45 °C), and weighed to determine soil dry weight (Precision balance: Sartorius GmbH) and carefully mortared to detach soil macroaggregates. The samples where then dry-sieved using covered stainless-steel sieves (Retsch GmbH) to the following size classes: >5 mm (macroplastic), >2 mm (coarse microplastic, coarse soil fraction) and <2 mm (fine-soil fraction)^47,48^. Following this procedure, 66 samples with a dry mass of 127.9–234.3 g were obtained. Soil sampling on direct neighbour and control sectors of field HB was not performed, as lateral microplastic distribution was investigated using the example of field LK only.

### Microplastic detection

Sieve fractions > 2 mm containing potential coarse microplastics or macroplastics were visually scanned^47^. For the fine soil fractions, a representative sub-set of 40 g dry soil material (<2 mm) of each sample, divided by means of a rotary sampler (Retsch GmbH), was analysed for microplastic content (plastic particles > 300 µm). For this purpose, two sub-sets of 20 ± 0.1 g of each respective sample were added to 30 ml of saturated and density-adjusted (ρ ≥1.20 g cm^3^, filtered >50 µm) NaCl solution (m:V 1:1.5), shaken by hand for one minute, and then centrifuged for 5 min at 3400 rpm (Megafuge 1.0, Retsch GmbH)^53^. The suspension was then rinsed with NaCl solution from the centrifuge tubes and sieved: >1000 µm and >300 µm (stainless-steel, Atechnik GmbH). Material smaller than 300 µm was discarded. The sieve residues were then cleaned with filtered deionized water and transferred to cellulose filters (LLG-Labware GmbH) via vacuum filtration and stored in glass petri dishes^54^. To distinguish between remaining organics and potential microplastics, the filters were stained with a Nile Red solution (20 μg mL^−1^ Nile Red, Sigma-Aldrich GmbH, dissolved in an ethanol-acetone (1:1) mixture) and then dried (50°C, 10 min)^55,56^. The stained filters were scanned under a stereomicroscope (SMZ 161 TL, Motic) with fluorescence setup (Excitation: 465 nm LED; Emissions: 530 nm color long pass filter; Thorlabs GmbH)^56^. Potential plastic particles were photo-imaged, their individual sizes measured (Moticam 2 and Motic Images Plus 3.0, Motic), and each plastic piece individually removed and stored in microplates (Brand, Wertheim, Germany). Potential plastic particles were counted as microplastics if they meet the criteria of non-organic particles^57^.

Due to the large number of existing (micro-)plastic analysis and separation methods^58^, the presented method was chosen to ensure the most efficient microplastic analysis for the investigated soils. To validate our method combination, we performed an additional concurrent testing: 5 self-produced microplastic particles (cut from industrial polymers with a density <1.2 g cm^3^, size: 300–1500 µm) were added to 36 separation runs (54.5% of all runs performed). The following common polymers were used: Polyethylene (HDPE and LDPE), Polypropylene (PP), Polyamides (PA), Polystyrene (PS) and Polycarbonates (PC). After the separation and staining procedure, we achieved an average recovery rate of 89.71% (HDPE: 92.0%, LDPE: 96.0%, PP: 88.9%, PA: 86.7%, PS: 86.7% and PC: 88.0%).

### Analyses

For identification of plastic items from surface sampling, a subset of 38.4% (total: 1075) of all items from field LK and 100% (total: 314) of items from field HB were analysed with a with a Tensor 37 FTIR spectrometer (BrukerOptics GmbH) combined with a Platinum-ATR-unit (BrukerOptics GmbH). Each item was measured after 16 background scans by 16 sample scans (spectral resolution: 4 cm^−1^ in a wavenumber range of 4000–400 cm^−1^). Spectra identification was carried out with the internal OPUS 7.0 (BrukerOptics GmbH) database, showing average OPUS-HIT ratios of 619.3 (LK) and 601.9 (HB). For microplastics from soil samples, a subset of 35.4% (total: 99) particles was analysed with a µFTIR spectrometer (Lumos II, BrukerOptics GmbH) with 30 background and 30 sample scans (spectral resolution: 4 cm^−1^ in a wavenumber range of 4000–680 cm^−1^). µFTIR spectra were identified using spectra correlation via OpenSpecy^59^, resulting in an average r^2^ of 0.84. Each plastic item or microplastic particle, regardless of the spectrometric analysis, was classified according to its visual surface characteristics (particle type, surface form and surface degradation)^60^. Statistical operations were performed in Microsoft Excel 2021 (Microsoft), and R (R Core Team, 2020), using RStudio (Version 3.4.1; RStudio Inc.). Spatial data analysis and processing was performed in QGIS^61^.

### Contamination prevention

During field work, only plastic-free devices were used. During further analysis, cotton laboratory coats were worn, and only plastic free equipment used. Laboratory equipment was rinsed with filtered (>50 µm) water, and only filtered (>50 µm) separation solution applied. Five control samples were analysed through all steps in which no plastic particles >300 µm were detected.

## Supplementary Information

Supplementary Information.

## Data Availability

The datasets generated and/or analysed during the current study are available in the **“**Research data of macro- and microplastics on agricultural fields 30 years after sewage sludge application” repository, https://doi.org/10.6084/m9.figshare.19312022.v1.
