# Supplementary material for: Investigating the dispersal of macro- and microplastics on agricultural fields 30 years after sewage sludge application
Source: Sci Rep. 2022 Apr 16;12:6401. doi: 10.1038/s41598-022-10294-w (PMC9013367; doi:10.1038/s41598-022-10294-w)
Supplement: Supplementary file 1 — Supplementary Information. [file 41598_2022_10294_MOESM1_ESM.docx]

Supplementary Information

**Investigating the dispersal of macro- and microplastics on agricultural fields 30 years after sewage sludge application**

Collin J. Weber, Alexander Santowski and Peter Chifflard

Correspond to: Collin J. Weber ([collin.weber@geo.uni-marburg.de](mailto:collin.weber@geo.uni-marburg.de))


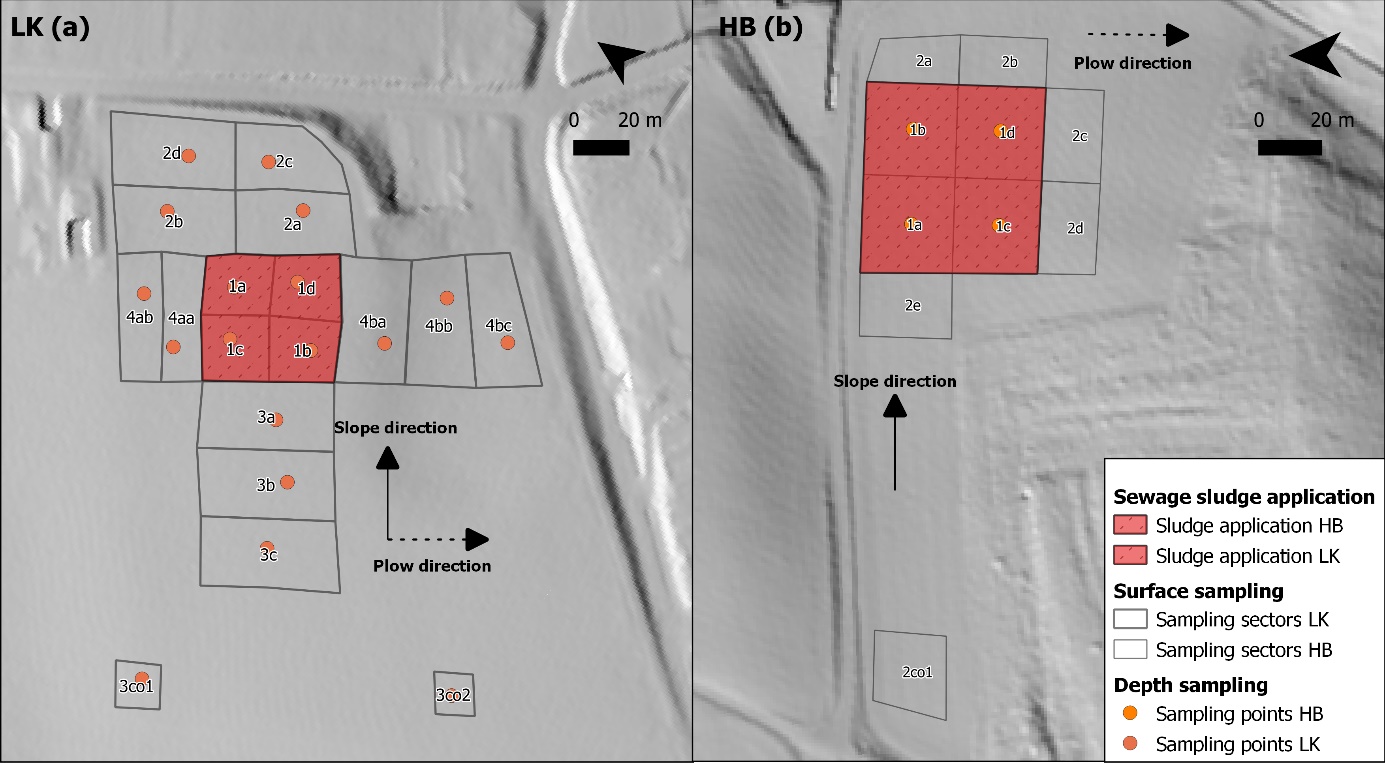


**Supplementary Figure S1.** Overview of sampling sectors for surface sampling and sampling point for depth sampling on agricultural fields **(a)** “Lehmkaute” (LK) and **(b)** “Holzbach” (HB) based on digital terrain model *(© Hessian State Office for Land Management and Geoinformation, 2019*) with slope and hang parallel plow directions.

**
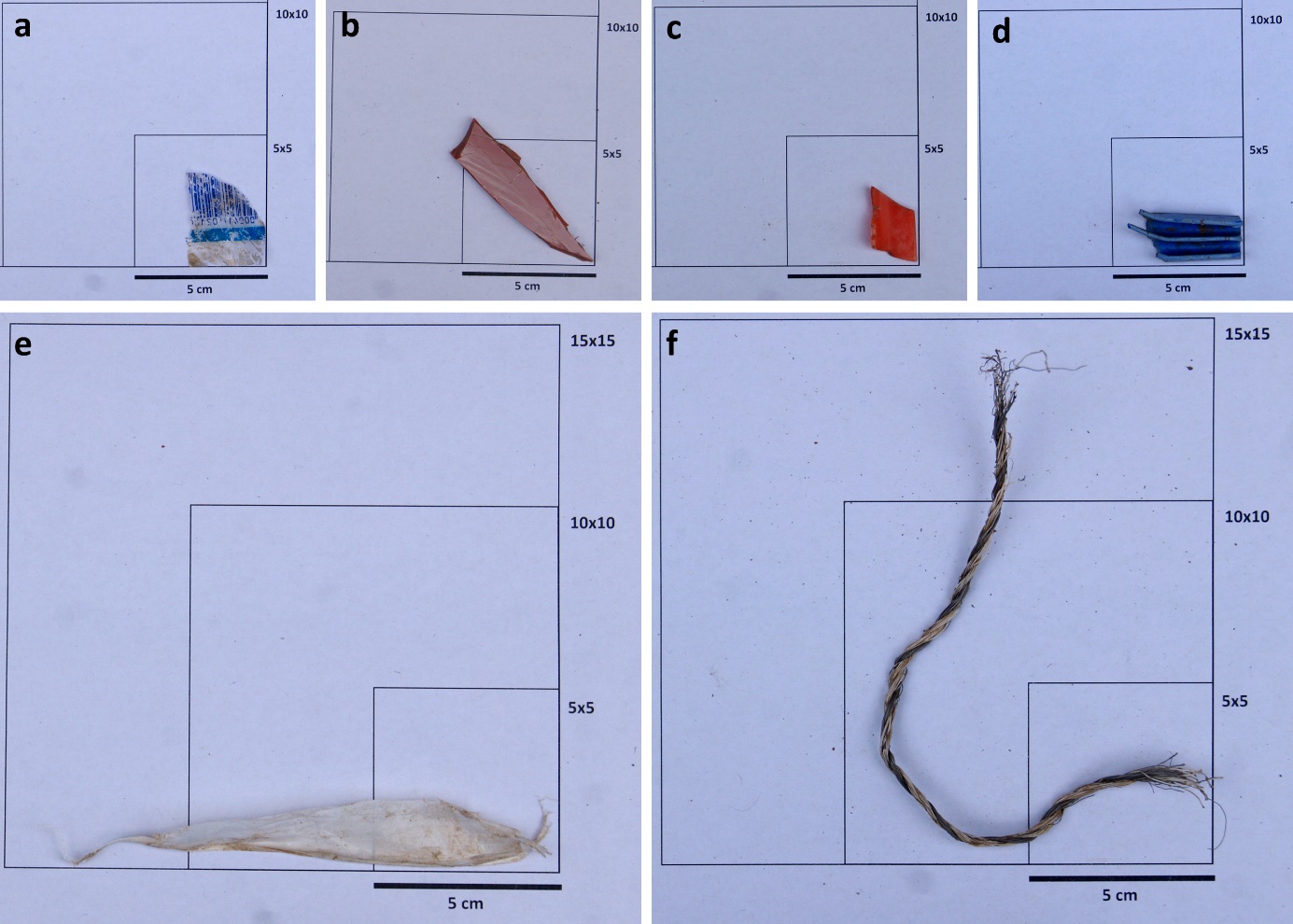
**

**Supplementary Figure S2**. Examples of collected plastic items on soil surfaces from field “Lehmkaute”showing **(a)** blue and white packaging residue made of polypropylene (PP), **(b)** red fragment made of polyvinyl chloride (PVC), **(c)** red fragment made of high-density polyethylene (HDPE), **(d)** blue fragment made of polypropylene (PP), **(e)** transparent film made of polyethylene terephthalate (PET) and **(f)** cable tie made of polyester fibres.


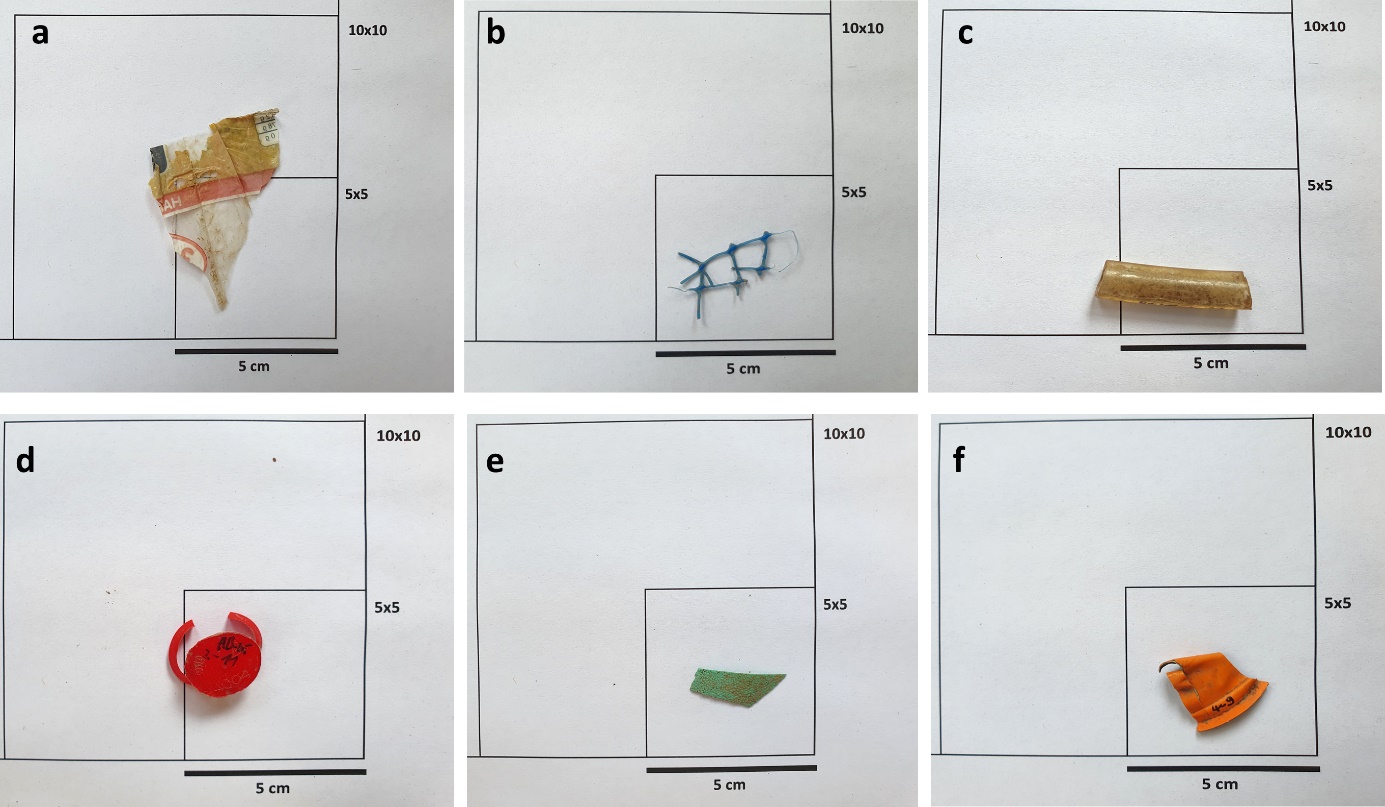


**Supplementary Figure S3**. Examples of collected plastic items on soil surfaces from field “Lehmkaute” showing **(a)** colored packaging residue made of polypropylene (PP), **(b)** blue net made of light-density polyethylene (LDPE), **(c)** cable fragment made of polyvinyl chloride (PVC), **(d)** red closure fragment made of light-density polyethylene (LDPE), **(e)** green fragment made of light-density polyethylene (LDPE) and **(f)** orange fragment made of polystyrene (PS).

**Supplementary Table S1:** Sewage sludge, management practice since 2016 and sampling area information.

|  | **LK ("Lehmkaute")** | **HB ("Holzbach")** |
| --- | --- | --- |
| Sludge type | Sewage sludge (yearly) and waste sewage sludge compost (3-year interval) with 22.8 % foreign matter (1981) | Sewage sludge (3-year interval) without data about foreign matter content |
| Sludge origin | Composting plant Bad Kreuznach (Rhineland-Palatinate) | Cappel wastewater treatment plant (Marburg, Hesse, Germany) |
| Agricultural crops | Beets (2016), Wheat (2017), Corn (2018), Wheat (2019), Beets (2020) | Rape (2016), Wheat (2017), Field grass (2018), Fallow (2019), Wheat (2020) |
| Organic fertilization | 89 m³ ha digestate in 2016, 2017 and 2020 | 45 m³ ha digestate in 2017 and 2020 |
| Further fertilizer applications | Mineral and mixed fertilizers (yearly), crop protection products | Mineral and mixed fertilizers (yearly), crop protection products |
| Sludge application duration | 1973-1986 | 1969-1986 |
| Sludge application rate | 120–200 t ha (3 year interval) | 2.5–5 t ha (3 year interval) |
| Sludge application area (m²) | 2313.2 | 3477.2 |
| Total field area (ha) | 5.69 | 1.71 |
| Investigated area (ha) | 1.51 | 0.67 |
| Number of sectors (drill cores) | 18 (18) | 10 (4) |
| Average sector size (m²) | 837.13 | 667.89 |

**Supplementary Table S2:** Weather conditions in the surroundings of the study area for the period 1986-2021 (after sewage sludge application)

|  | **Period 1986-2021** | | |
| --- | --- | --- | --- |
|  | Min | Average | Max |
| Annual average temperature (°C) | 7.6 | 9.7 | 10.8 |
| Annual temperature deviation (°C) | -2.1 | -0.1 | 1.1 |
| Precipitation annual sum (mm) | 543.0 | 678.5 | 873.7 |
| Days with snow cover (>1 cm) | 0.0 | 17.7 | 58.0 |
| Frost days | 51.0 | 78.4 | 117.0 |
| *Data source: Cölbe weather station (ID: DWD 3164) 50.8489°N, 8.7733°E. Distance from the study area: 12 km (Data obtained through: https://www.hlnug.de/messwerte/witterungs-und-klimadaten/wetterextreme)* | | | |

**Supplementary Table S3:** Wind conditions in the surroundings of the study area for the period 1986-2021 showing 10 years interval (after sewage sludge application)

| Year | Average annual wind speed (km h^-1^) | Average maximal gust speed (km h^-1^) |
| --- | --- | --- |
| 1986 | 9.67 | 72.00 |
| 1996 | 8.58 | 61.42 |
| 2006 | 11.33 | 67.67 |
| 2016 | 11.08 | 65.08 |
| 2020 | 11.67 | 65.75 |
| *Data source: Gießen weather station (ID: DWD 1639) 50.6014°N, 8.6439°E. Distance from the study area: 20 km (Data obtained through: https://www.hlnug.de/messwerte/witterungs-und-klimadaten/wetterextreme)* | | |
